# IgA nephropathy in Brazil: apropos of 600 cases

**DOI:** 10.1186/s40064-015-1323-x

**Published:** 2015-09-24

**Authors:** Maria Fernanda Soares, M. L. R. Caldas, W. L. C. Dos-Santos, A. Sementilli, P. Furtado, S. Araújo, K. L. Pegas, R. R. Petterle, M. F. Franco

**Affiliations:** Department of Pathology, Federal University of São Paulo (UNIFESP), São Paulo, Brazil; Department of Medical Pathology, Federal University of Paraná (UFPR), Curitiba, Brazil; Department of Pathology, Federal Fluminense University (UFF), Niterói, Brazil; Centro de Pesquisas Gonçalo Moniz, Oswaldo Cruz Foundation (CPqGM-FIOCRUZ), Salvador, Brazil; Santos Metropolitan University (UNIMES) and Ana Costa Hospital, Santos, Brazil; Clinics Hospital, Federal University of Minas Gerais (UFMG), Belo Horizonte, Brazil; Federal University of Ouro Preto (UFOP), Ouro Prêto, Brazil; Santa Casa de Misericórdia de Porto Alegre, Porto Alegre, Brazil; Brazilian Kidney Club, Brazilian Society of Pathology (SBP), São Paulo, Brazil; Division of Health Sciences, Federal University of Parana (UFPR), Curitiba, Brazil; Departamento de Patologia Médica, Rua Padre Camargo, 280, 6. andar, Curitiba, PR 80060-240 Brazil

**Keywords:** IgA nephropathy, Berger’s disease, Pathology, Epidemiology

## Abstract

IgA nephropathy (IgAN) is th e commonest primary glomerular disease worldwide. Studies on its prevalence in Brazil are however scarce. Databases and clinical records from 10 reference centres were retrospectively reviewed. Clinical and laboratory features at the moment of the biopsy were retrieved (age, gender, presence of hematuria, serum creatinine [mg/dL], proteinuria [g/24 h]). Renal biopsy findings were classified according to Haas single grade classification scheme and the Oxford Classification of IgAN. 600 cases of IgAN were identified, of which 568 (94.7 %) were on native kidneys. Male to female ratio was 1.24:1. Patients averaged 32.76 ± 15.12 years old (range 4–89, median 32). Proteinuria and hematuria were observed, respectively in 56.63 and 72.29 % of patients. The association of both these findings occurred in 37.95 % of the cases. Serum creatinine averaged 1.65 ± 0.67 mg/dL (median 1.5 mg/dL) at diagnosis. Segmental sclerosis and mesangial hypercellularity were the main glomerular findings (47.6 and 46.2 %) The commonest combination by Oxford Classification of IgAN, was M0 E0 S0 T0 (22.4 %). Chronic tubulo-interstitial lesions with an extension wider than 25 % of the renal cortex could be identified in 32.2 % of the cases. Tubular atrophy and interstitial fibrosis were more strongly associated with higher 24-h proteinuria and serum creatinine levels. Segmental sclerosis (S1) showed a stronger tendency of association with the presence of tubulo-interstitial lesions (T1 and T2) than other glomerular variables. To the best of our knowledge this is the largest series of IgAN in Brazil. It depicts the main biopsy findings and their possible clinical correlates. Our set of data is comparable to previous reports.

## Background

IgA nephropathy (IgAN) is the primary glomerular disease with the highest prevalence in the world (Roberts 2014; Wyatt and Julian 2013). From the pathologist’s viewpoint, the diagnosis of IgAN is relatively straightforward, through the identification of dominant or codominant immunoglobulin A (IgA) deposits of mesangial and, to a lesser degree, capillary wall location by immunofluorescence or immunoperoxidase techniques. Despite the relative objectivity of the immunofluorescence/immunoperoxidase diagnostic criteria, light microscopy changes tend to be variable, ranging from normality to severe proliferative and chronic scleroatrophic lesions, which reflect the diversity of the clinical picture of the disease (Roberts 2014; Wyatt and Julian 2013; Haas 2007; Hennigar and Tumlin 2009). From the clinician viewpoint, a number of clinical and laboratory parameters have been correlated with worse clinical outcomes, such as the rise of serum activated C3, elevated urinary interleukin-6 (IL-6) level and the increased relation of urinary IL-6 and epidermal growth factor (EGF) (Zwirner et al. 1997; Ranieri et al. 1996). In clinical practice, however, usual prognostic factors in IgAN are glomerular filtration rate, serum creatinine value and 24-h proteinuria (Barratt and Feehally 2011).

IgAN is regarded as the commonest primary glomerulonephritis in most developed countries (Roberts 2014; Wyatt and Julian 2013). The male to female ratio is 2:1 in North American and Western European cohorts, with a peak incidence in the second and third decades of life (Wyatt and Julian 2013). A higher frequency of the disease is observed among Asian populations, but this finding may reflect different biopsy recommendations and screening policies (Roberts 2014). Figures on the prevalence and impact of IgAN in Brazil are however scarce. A recent paper regarding 9617 countrywide renal biopsies depicted a 9.6 % and a 20.1 % incidence among all and among primary glomerulopathies, respectively (Polito et al. 2009). IgAN accounts for varying ranges of 1–10.7 % of all glomerulopathies and 2–25 % of primary glomerulopathies according to other local reports (Ferraz 2010; Queiroz 2009; Cardozo and Kirsztajn 2006). Besides, data on the correlation of histopathological findings to clinical outcome are lacking in our country.

## Methods

This study was conducted under the auspices of the Kidney Club of the Brazilian Society of Pathology. Twelve renal pathologists pertaining to ten institutions contributed with data (UNIFESP, 108 cases; UFPR, 44 cases; UFF, 36 cases; UFOP and UFMG, 55 cases; Ana Costa Hospital, 9 cases; UNICAMP, 241 cases; CPqGM-FIOCRUZ, 17 cases; UFMA, 26 cases; Santa Casa de Misericordia de Porto Alegre, 64 cases).

Renal tissue was obtained by biopsy and processed for light microscopy, immunofluorescence microscopy, with a panel of anti-human antibodies directed towards IgA, IgG, IgM, C3, C1q and kappa and lambda light chains, and electron microscopy when available. IgAN was defined as the finding of consistent dominant granular to globular IgA deposits in mesangial areas and less prominently along capillary walls or codominant deposits with the same distribution pattern alongside milder C3 or IgG deposits. Light microscopy findings were classified by Haas single grade classification scheme (Haas 2007) and by Oxford Classification of IgAN (Working Group of the International IgA Nephropathy Network and the Renal Pathology Society et al. 2009a, b). Clinical data—24 h proteinuria, serum creatinine and main clinical features at the moment of the biopsy were collected from clinical reports when available. Descriptive statistical data were expressed as means and standard deviations. Inferential statistics was performed through Wilcoxon rank sum test with continuity correction, Kruskal–Wallis rank sum test and Fisher’s exact test.

## Results

Six hundred (600) consecutive cases were identified from 2006 to 2013 in ten Brazilian reference centres of which 568 (94.7 %) were reported on native kidneys and 32 (5.3 %) were reported on transplanted kidneys. Male to female ratio was 1.24:1. Patients averaged 32.67 ± 15.12 years old (range 4–89; median 32). Hematuria was present in 56.63 % of patients, which was isolated in 27.66 % of these patients. Proteinuria was observed in 72.29 % of patients, which was within the nephrotic range in 28 % of these. Frank nephrotic syndrome was observed in 21 % of the patients with proteinuria. Overlapping proteinuria and hematuria was observed in 37.95 % of the patients in the cohort. Proteinuria levels averaged 3.27 ± 3.13 g/24 h (range 0.19–12; median 2.0). Average serum creatine value at biopsy was of 1.65 ± 0.67 mg/dL (range 0.8–3.75; median 1.5). Other presentations included rapidly progressive glomerulonephritis (2.4 %) and nephritic syndrome (1.2 %).

Regarding pathological features, all cases (100 %) depicted diffuse and dominant mesangial IgA deposits with moderate to absent concurrent C3 deposits at immunofluorescence microscopy, in keeping with the diagnosis of IgAN. In 300 cases (50 %) electron microscopy was available and revealed variable mesangial to paramesangial electrondense deposits. 508 cases (85.23 %) showed signs of glomerular lesions at light microscopy. Segmental sclerosis (Haas class II, Oxford S1), mesangial hypercellularity (Haas class I, Oxford M1) and endocapillary proliferation (Haas classes III and IV, Oxford E1) were observed, respectively in 47.6, 42.2 and 13.7 % of the patients. Crescents were identified in 13.2 % of the cases of which 70.9 % were cellular and 29.1 % fibrocellular. Signs of advanced sclerosing glomerulonephritis (Haas class V) were present at diagnosis in 10.7 % of the patients. Glomerular lesions could not be adequately classified in 3 of the 346 cases (0.87 %) due to superimposed lesions of diabetic nephropathy. None of the transplanted kidneys depicted signs of transplant glomerulopathy.

Absent to mild tubular atrophy and interstitial fibrosis (Oxford T0) was identified in 67.8 % of the cases. Moderate (Oxford T1) and severe (Oxford T2) tubular atrophy and interstitial fibrosis were present at diagnosis in 20.7 and 11.5 % of the cases. Overall, the most prevalent pattern association according to the Oxford Classification was M0 E0 S0 T0 (22.4 %).

**Fig. 1:**
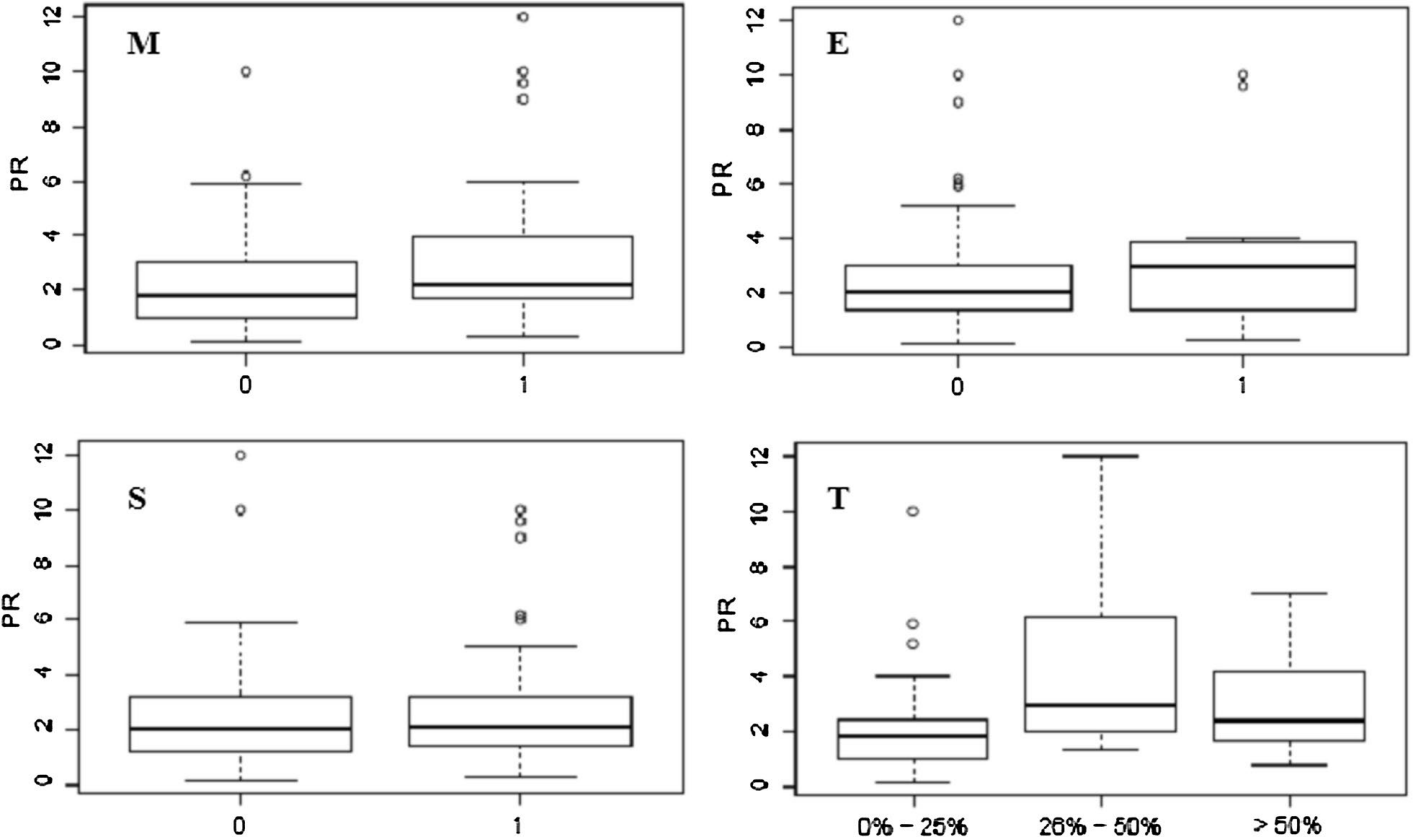
24-h proteinuria (g/24 h) values according to Oxford classification. *M* mesangial hypercellularity (*0* absent; *1* present); *E* endocapillary proliferation (*0* absent; *1* present); *S* segmental sclerosis/adhesions/synechiae (*0* absent; *1* present); *T* tubular atrophy/interstitial fibrosis (0–0–25 %; 1–26–50 %; 2 to >50 %)

**Fig. 2:**
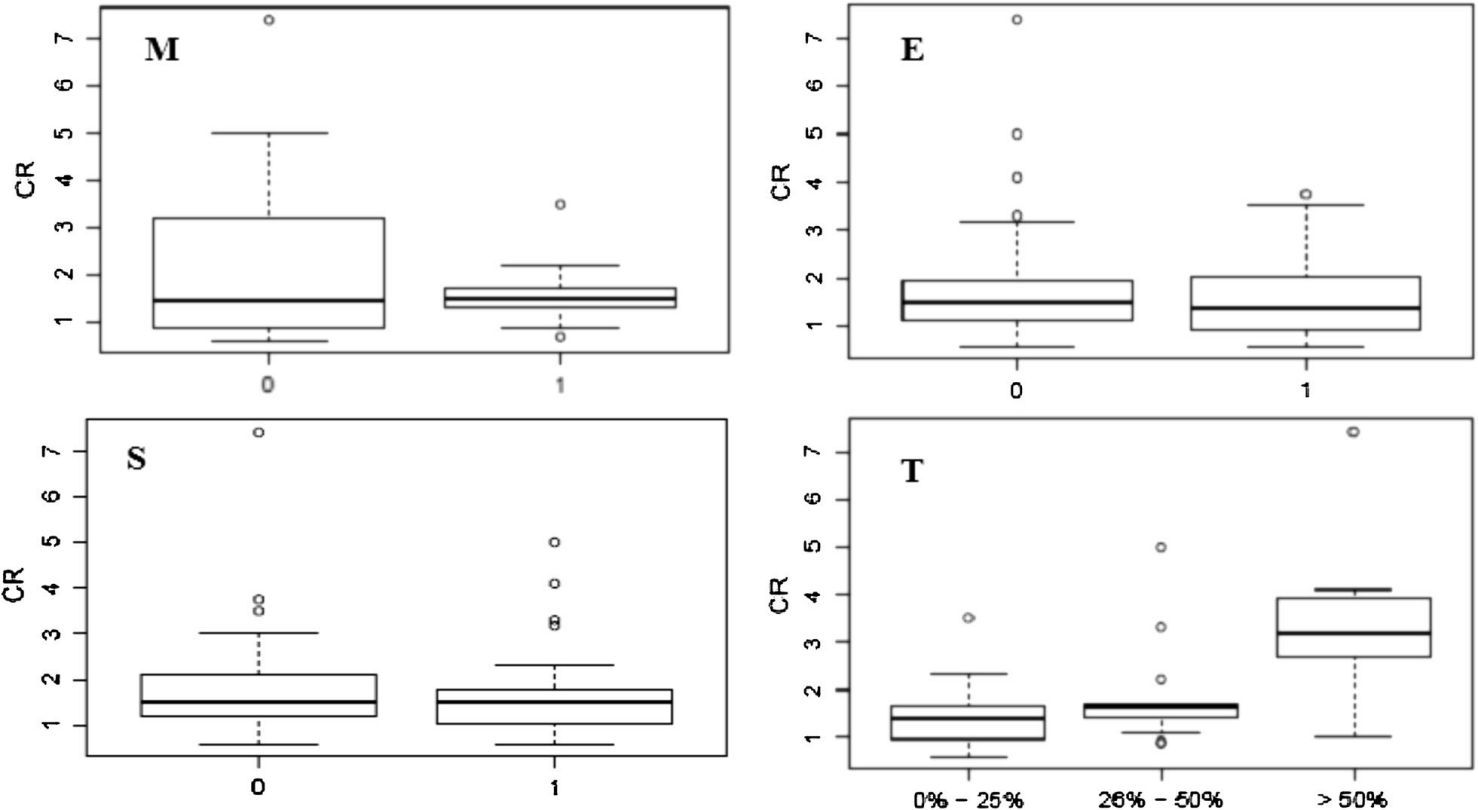
Serum creatinine (mg/dL) values according to Oxford classification. *M* mesangial hypercellularity (*0* absent; *1* present); *E* endocapillary proliferation (*0* absent; *1* present); *S* segmental sclerosis/adhesions/synechiae (*0* absent; *1* present); *T* tubular atrophy/interstitial fibrosis (0–0–25 %; 1–26–50 %; 2 to >50 %)

Wilcoxon rank sum test and Kruskal–Wallis rank sum test were performed to test if any of the Oxford classification variables were more strongly associated with laboratory data. A trend towards higher 24-h proteinuria levels was observed in patients whose biopsies revealed mesangial hypercellularity (Oxford M0 = 2.35 ± 2.08 g/24 h vs. Oxford M1 = 3.51 ± 2.99 g/24 h; P = 0.06). The presence of moderate tubular atrophy and interstitial fibrosis was associated with higher levels of 24-h proteinuria (Oxford T0 = 2.14 ± 1.85 g/24 h vs. Oxford T1 = 3.09 ± 2.66 g/24 h; P < 0.001). However, no significant differences were found regarding 24-h proteinuria levels when comparing Oxford T0 and T2 (3.02 ± 2.07 g/24 h) or Oxford T1 with T2 (P = 0.22 and 0.20) (Fig. 1). Regarding serum creatinine levels the more extensive the tubular atrophy and interstitial fibrosis the higher the serum creatinine levels (Oxford T0 = 1.45 ± 0.67 mg/dL; Oxford T1 = 1.61 ± 0.72 mg/dL; Oxford T2 = 3.55 ± 1.98 mg/dL; T0 vs. T2 P < 0.001; T1 vs. T2 P = 0.04). However patients with moderate tubular atrophy and interstitial fibrosis did not show higher serum creatinine levels when compared to patients with none to mild changes (Oxford T1 vs. T0, P = 0.13) (Fig. 2). The abovementioned results indicate that tubulo-interstitial but not glomerular variables were associated with worst laboratory parameters—24-h proteinuria and higher serum creatinine levels. We found out that when all glomerular variables equaled zero (M0, E0 and S0), T equaled zero (T0) in a significantly higher frequency than that of T1 or T2 cases (22.38 vs. 6.39 %, P = 4.475e−09). Thus, to test if any single glomerular variable was more frequently associated with the presence of chronic tubulo-interstitial lesions (T ≠ 0) we carried out Fisher’s exact test. None of the glomerular variables was more frequently associated to the presence of tubular atrophy and interstitial fibrosis (Oxford T1 and T2). However upon the presence of segmental sclerosis (S1) there was a slight trend towards a higher frequency of T1 and T2 lesions (M0 E0 S1 T0 = 14.8 %; M0 E0 S1 T ≠ 0 = 7.8 %; P = 0.09). This trend could not be proved for M1 and E1 lesions (M1 E0 S0 T0 = 11.9 %; M1 E0 S0 T ≠ 0 = 3.8 %; P = 0.84/M0 E1 S0 T0 = 0.9 %; M0 E1 S0 T ≠ 0 = 0.9 %; P = 0.15). These figures are summarised on Table 1.

**Table 1:** Frequency of chronic tubulointerstitial lesions (Oxford T1 and T2) according to glomerular variables

Oxford classification	Frequency (%)	P (Fisher’s exact test)
M1 E0 S0 T0	11.9	0.84
M1 E0 S0 T ≠ 0	3.8	
M0 E1 S0 T0	0.9	0.15
M0 E1 S0 T ≠ 0	0.9	
M0 E0 S1 T0	14.8	0.09
M0 E0 S1 T ≠ 0	7.8	

## Discussion

This study presents the largest IgAN cohort to date in this particular South American country. It encompasses kidney samples obtained in 10 reference institutions located on the three most densely populated and ethnically diverse areas of the country (Northeast, 2 centres, 34 cases; Southeast, 6 centres, 458 cases; South, 2 centres, 108 cases). IgAN patients from—have a slight male predominance (55.4 %) as opposed to the Oxford Classification original cohort (72 %) (Working Group of the International IgA Nephropathy Network and the Renal Pathology Society et al. 2009a; b) and North American data (64 %) (Wyatt and Julian 2013) but in accordance with a Chinese (50 %) (Zeng et al. 2012) and a previous national cohort (53.7 %) (Neves et al. 2012). Patients were diagnosed with IgAN at the average age of 32.67 years old similarly as other reported series including the Oxford Classification cohort (Working Group of the International IgA Nephropathy Network and the Renal Pathology Society et al. 2009b; Zeng et al. 2012). Reported incidence of hematuria (56.63 %) is in dissonance with a large recent Chinese series (27 %) (Zeng et al. 2012). However Wyatt and Julian (2013) reported hematuria during upper respiratory tract or gastrointestinal tract disease in 75 % of the North American patients. In this cohort patients presented at diagnosis with average 24-h proteinuria levels near the nephrotic range (3.27 g/24 h), higher than previous reports (Zeng et al. = 1.3 g; Oxford = 1.7 g; Neves = 1.8 g; Moriyama = 1.19 g) (Working Group of the International IgA Nephropathy Network and the Renal Pathology Society et al. 2009a, b; Zeng et al. 2012; Neves et al. 2012; Moriyama et al. 2014) but comparable to the data by Huerta et al. (2011) (3.7 g). However the higher levels of proteinuria in our series and the frequency of nephrotic syndrome as initial presentation could be a result of biopsy indication bias. Unfortunately, none of the included cases with massive, nephrotic range proteinuria, underwent ultrastructural studies and superimposed podocytopathy/minimal change disease could not be ruled out. 24-h proteinuria might thus be not completely attributable to IgAN in these particular cases.

The importance of proteinuria as a predictor of decreasing estimated glomerular filtration rate (eGFR) was highlighted by previous studies (Moriyama et al. 2014; Bartosik et al. 2001; Barbour and Reich 2012). Thus maintenance of 24-h proteinuria levels below 1 g/24 h is one of the targets in the management of IgAN. Recognising which pathological variables are associated with ensuing proteinuria is hence a useful piece of information. The original Oxford Classification cohort demonstrated that mesangial hypercellularity, segmental sclerosis and endocapillary proliferation were histopathological variables associated with proteinuria (Working Group of the International IgA Nephropathy Network and the Renal Pathology Society et al. 2009b). Indeed, a trend towards higher 24-h proteinuria levels was observed in patients with mesangial hypercellularity (M1) which is similar to previous observations in Brazil, South Korea and Sweden (Neves et al. 2012; Kang et al. 2012; Halling et al. 2012). This series however demonstrated that migration from a T0 to a T1 pattern was also associated with proteinuria. Increasing extent of interstitial fibrosis and tubular atrophy was also a marker of rising serum creatinine levels, in keeping with the seminal findings of the Oxford Classification cohort. These data underline the importance of tubulo-interstitial lesions in IgAN as opposed to the relative underestimation of this compartment in other classification schemes applied to IgAN and to other diseases which overemphasise the importance of glomerular pathology. We thus tried to pinpoint which glomerular variables were more strongly associated with T1 and T2 lesions. Only the presence segmental sclerosis, adhesions and synechiae (S1 lesions) seem to be somewhat associated with chronic tubular and interstitial impairment. This finding is partly supported by previous reports which point out a possible podocytopathic pathway and the involvement of multiple glomerular structures in the development of focal segmental sclerosis lesions in IgAN. These findings alongside our data may support recent evidence that suggest that mesangial IgA deposition triggers podocyte lesion and the development of adhesions and synechiae which in turn leads to tubulo-interstitial injury via podocyte-tubular crosstalk (Menon et al. 2013; Wang et al. 2012; Lai 2012; Zhu et al. 2013). These phenomena may yield epithelial-mesenchymal transition and consequent interstitial fibrogenesis.

## Conclusions

To date this is the largest series of IgAN in this country. Demographic data were consistent with previous published reports—slight male predominance of patients in their 30 s, presenting with hematuria and proteinuria and slightly elevated serum creatinine at diagnosis. M1 and T1 lesions were more strongly associated with proteinuria. Higher serum creatinine values were identified in patients with T1 and T2 lesions. S1 lesions tended to be more closely associated with the development of chronic tubulo-interstitial injury. Despite its shortcomings—lack of full ultrastructural studies for all cases, absence of follow up data and scarce clinical data—this study displays a large series of cases stemming from a nationwide database. Further investigation regarding the role of crescents, reproducibility of diagnostic criteria and validation of the Oxford classification in this country are still pending.
